# Predicting the orientation of protein G B1 on hydrophobic surfaces using Monte Carlo simulations

**DOI:** 10.1116/1.4971381

**Published:** 2016-12-06

**Authors:** Elisa T. Harrison, Tobias Weidner, David G. Castner, Gianluca Interlandi

**Affiliations:** Department of Chemical Engineering, University of Washington, Seattle, Washington 98195; Department of Chemistry, Aarhus University, 8000 Aarhus C, Denmark and Max Planck Institute for Polymer Research, 55128 Mainz, Germany; Department of Chemical Engineering, University of Washington, Seattle, Washington 98195 and Department of Bioengineering, University of Washington, Seattle, Washington 98195; Department of Bioengineering, University of Washington, Seattle, Washington 98195

## Abstract

A Monte Carlo algorithm was developed to predict the most likely orientations of protein G B1, an immunoglobulin G (IgG) antibody-binding domain of protein G, adsorbed onto a hydrophobic surface. At each Monte Carlo step, the protein was rotated and translated as a rigid body. The assumption about rigidity was supported by quartz crystal microbalance with dissipation monitoring experiments, which indicated that protein G B1 adsorbed on a polystyrene surface with its native structure conserved and showed that its IgG antibody-binding activity was retained. The Monte Carlo simulations predicted that protein G B1 is likely adsorbed onto a hydrophobic surface in two different orientations, characterized as two mutually exclusive sets of amino acids contacting the surface. This was consistent with sum frequency generation (SFG) vibrational spectroscopy results. In fact, theoretical SFG spectra calculated from an equal combination of the two predicted orientations exhibited reasonable agreement with measured spectra of protein G B1 on polystyrene surfaces. Also, in explicit solvent molecular dynamics simulations, protein G B1 maintained its predicted orientation in three out of four runs. This work shows that using a Monte Carlo approach can provide an accurate estimate of a protein orientation on a hydrophobic surface, which complements experimental surface analysis techniques and provides an initial system to study the interaction between a protein and a surface in molecular dynamics simulations.

## I. INTRODUCTION

Materials in contact with the biological environment will immediately become coated with proteins and this adsorbed protein layer thus becomes the interface between the material and species in the surrounding environment, such as proteins, antibodies, antigens, lipids, and cells.^1,2^ The orientation of the adsorbed proteins may influence the interaction of the material with the biological environment.^3^ For example, exposing active sites of binding proteins may increase efficiencies of *in vitro* diagnostic devices such as enzyme-linked immunosorbent assays (ELISAs).^4^ However, one main challenge for developing and improving devices like ELISA is determining the conformation and orientation of the proteins when adsorbed onto a surface, especially when multicomponent protein films are present. In fact, besides the possibility of undergoing conformational changes, protein molecules adsorbed onto surfaces might also align in an ordered manner with respect to the surface. In this manuscript, the orientation of a protein is characterized by which amino acids contact the surface and by the angle between the two major axes of symmetry and the surface normal. As the need for the improvement and the complexity of devices such as ELISA increases, so does the need for the development of techniques that can provide a complete understanding of protein–surface interactions. This includes characterizing both, how proteins are ordered (i.e., their orientation with respect to the surface) and any conformational changes the proteins might undergo upon adsorption.

Proteins immobilized onto surfaces have been extensively studied using experimental methods.^5–11^ Techniques such as quartz crystal microbalance with dissipation monitoring (QCM-D), surface plasmon resonance, and ellipsometry can provide useful information such as binding efficiencies and kinetics of adsorbing proteins, but can only indirectly suggest orientation information since many other factors influence protein binding.^3,12^ The surface sensitivity and the chemical specificity of time-of-flight secondary ion mass spectrometry (ToF-SIMS) can provide conformation and orientation information by tracking changes in secondary ion intensities from amino acid fragments unevenly distributed throughout the protein.^13–15^ However, while this technique can provide orientation information, the conformation and structure of immobilized proteins may change upon being dried and subjecting them to ultrahigh vacuum prior to analysis. Sum frequency generation (SFG) vibrational spectroscopy provides orientation information since it relies on order within the sample, and samples can be analyzed in solution.^16–18^ However, many of these techniques require that all proteins are uniformly oriented on the surface and are limited to providing an average orientation. For example, if multiple orientations of the protein exist on a given surface, extracting the different orientations from the experimental data becomes increasingly difficult as the number of orientations increases.

To complement and more completely interpret experimental results, the development of computational methods is needed to predict the structure of proteins on surfaces. Classical molecular dynamics (MD) simulations can provide atomic-level information on interactions between proteins and surfaces that may not be accessible with experimental techniques.^19,20^ However, because of the vast conformational space and large number of degrees of freedom present when describing protein–surface interactions, classical MD simulations are limited to time scales that may not be long enough to sample all protein orientations on the surface. The Monte Carlo-based method, Rosetta, has been developed to efficiently sample the conformational space of a protein,^21^ and it has been recently applied to study the interaction between the protein statherin and hydroxyapatite.^22^ However, Rosetta relies on a knowledge-based scoring function, and currently not enough examples of protein–surface systems have been characterized at the level of detail required to correctly optimize Rosetta's scoring function. Thus, there is the necessity to develop approaches that do not rely on the existence of fully characterized protein–surface models.

In this work, we have developed a method to predict the orientation of proteins on surfaces based on Monte Carlo simulations and an implicit solvation model. The developed algorithm was applied to determine the orientation of the LKα14 peptide and protein G B1 on a graphene surface. The LKα14 peptide is a 14 amino-acid model peptide consisting of only leucine and lysine residues that was designed to form an α-helical secondary structure when immobilized onto either hydrophobic or hydrophilic surfaces.^23^ Since its orientation on surfaces is known, it was used here as a benchmark. The second, more complex system is protein G B1, a 6 kDa domain of protein G which binds to the Fc region of immunoglobulin (IgG) antibodies. This protein was chosen for this study because of its stability, both in solution and immobilized on surfaces, and the availability of experimental data.^24^ The predictions from Monte Carlo simulations with protein G B1 were validated through SFG experiments. Finally, MD simulations in explicit solvent investigated the conformational stability of protein G B1 on graphene surfaces. The present work highlights how Monte Carlo simulations allow the sampling of protein orientations on surfaces at a lower computational cost than conventional MD simulations. The predictions can then be used to complement results from surface analysis experiments that do not provide atomic level detail or to start explicit solvent MD simulations to study conformational changes of the protein at the interface with a surface.

## II. MATERIALS AND METHODS

### A. Monte Carlo simulations

The Monte Carlo simulations performed here are based on the Metropolis criterion.^25,26^ At each Monte Carlo step, the protein was randomly translated and rotated as a rigid body while the surface was kept frozen. Periodic boundary conditions were applied to simulate an infinite surface. After a move, the total energy of the system was evaluated, and the move was accepted or rejected according to the Metropolis criterion.^25,26^ The accepted orientations thus satisfy the Boltzmann distribution. The total energy of the system consisted of a van der Waals (VDW) interaction term and a solvation term to account for the hydrophobic effect. The VDW energy term was calculated using the program gromacs 4.6 (Ref. 27) with the CHARMM22 force field.^28^ Electrostatic interactions did not need to be calculated because graphene consists of only carbon atoms with neutral charges. Future versions of the algorithm that include surfaces with polar groups will need to consider also electrostatic interactions.

To take solvation effects into account, we introduced a nonpolar solvation term based on the solvent accessible surface area (SASA). This allowed us to penalize orientations where hydrophobic atoms, in both the protein and surface, are exposed to the solvent.^29^ The nonpolar solvation energy term was calculated by multiplying a surface tension parameter (σ) by the SASA of the groups of atoms listed in Table I. The SASA was calculated using a 1.4 Å probing radius by means of the gromacs package.

**Table I.** Surface tension parameters (σ) used in the evaluation of the nonpolar solvation term.

Group	Description	σ (kJ/mol Å^2^)
Hydrophobic sidechain	Residue name: Gly, Ala, Val, Leu, Ile, Met, Pro, Phe, Trp, and the aromatic ring of Tyr	100
Hydrophilic sidechain	Residue name: Ser, Thr, Asn, Gln, Cys, Arg, Asp, His, Lys, Glu, and the hydroxyl group of Tyr	−100
Hydrophobic backbone atoms	Cα-H and carbonyl carbon (C)	100
Hydrophilic backbone atoms	Carbonyl oxygen (O) and amide group (N-H)	−100
Hydrophobic surface atoms	Carbon (C)	100

The script is written in toolkit command language and runs using the program visual molecular dynamics (vmd).^30^ The crystallographic structure of protein G B1 (PDB code 1PGA) was used. Prior to the Monte Carlo simulation, the coordinates of the protein were minimized in a generalized Born implicit solvent^31,32^ with 1000 steps of steepest descent by means of the program gromacs.^27^ Three Monte Carlo simulations were run for the LKα14 peptide and 13 Monte Carlo simulations were run for protein G B1. Each simulation was run for a total of 10 000 accepted moves, during which all but one simulation with protein G B1 converged to a stable orientation.

### B. Molecular dynamics simulations

The MD simulations were performed using the program gromacs 4.6 (Ref. 27) with the CHARMM22 force field^28^ containing the correction map (CMAP) extension^33,34^ (this is generally referred to as the CHARMM22/CMAP force field). Although the force field used here is known to overestimate the stability of α-helical structures, we do not expect a significantly different outcome of the simulations even when using the latest available CHARMM36 force field, where this issue has been corrected,^35^ since the protein is shown here to be rigid when adsorbed. The protein G B1 and the graphene surface were placed in a 47.96 × 42.6 × 70 Å^3^ water box containing 3666 TIP3P water molecules and four Na^+^ ions. The system was minimized using 1000 steps of steepest descent. After minimization, positional restraints were applied to heavy atoms (all atoms excluding hydrogen atoms) of the protein and the system was simulated for 200 ps, during which the temperature of the system was ramped from 5 to 300 K and the water molecules equilibrated around the surface of the protein.

After this equilibration phase, the positional restraints were released, and the system was simulated for a total of 50 ns. During the dynamics, the translation of the center of mass was removed, and three-dimensional periodic boundary conditions were applied. The graphene surface atoms were kept fixed during the dynamics. Electrostatic and van der Waals interactions were calculated within a cutoff of 10 Å while long range electrostatic effects were taken into account by the particle mesh Ewald summation method.^36^ The Nosé-Hoover thermostat^37,38^ with a time constant of 0.4 ps was used to maintain the temperature at 300 K. All bonds involving hydrogen atoms were kept fixed using the LINear Constraint Solver algorithm.^39^ The dynamics were integrated with a time step of 2 fs, and snapshots were saved every 10 ps.

### C. Quartz crystal microbalance with dissipation monitoring

Adsorption and binding of protein G B1 and IgG antibody onto polystyrene surfaces was monitored using the E4 QCM-D (Q-Sense, Sweden) system. Frequency and dissipation measurements were made on polystyrene-coated gold quartz crystals with fundamental frequencies of 4.95 MHz (Q-Sense, Sweden). The analysis for these measurements used the fifth and seventh overtone. Experiments were repeated three times. The temperature was maintained at 22 °C.

During the course of each QCM-D experiment, protein G B1 was adsorbed onto the polystyrene-coated gold sensors from a 5 *μ*g/ml phosphate buffered saline (PBS) solution (0.01 M phosphate, 0.138 M sodium chloride, and 0.0027 M potassium chloride) at a flow rate of 150 *μ*l/min and a *p*H of 7.4. Following protein G B1 adsorption, the system was rinsed with PBS buffer at a 150 *μ*l/min flow rate to remove any excess protein. IgG antibodies were then immobilized to adsorbed protein G B1 from a 5 *μ*g/ml PBS solution at a flow rate of 150 *μ*l/min and a *p*H of 7.4. Again, the system was rinsed with PBS buffer at a 150 *μ*l/min flow rate to remove unbound antibodies. Dissipation was monitored to ensure that protein G B1 did not undergo significant viscoelastic changes upon adsorption.

### D. Sum frequency generation spectroscopy

Details of the SFG setup are published elsewhere and will only be briefly discussed here.^40^ The visible beam from an EKSPLA Nd:YAG laser with a wavelength of 532 nm and the tunable IR beam from an EKSPLA optical parametric generation/amplification unit were focused at the sample with energies of 120 and 180 *μ*J per pulse for the visible and the IR beams, respectively. The spectra were collected with 200 shots per data point in 4 cm^−1^ increments. The SFG spectra were normalized by the product of the intensities of the IR and visible pump beams, which were tracked with photodiodes. The input angles of the visible and IR beams after entering the prism were 47° and 58° with respect to the surface normal.

One side of the CaF_2_ prism was spin coated with a ∼100 nm polystyrene film according to a procedure described in Ref. 40. The polystyrene side was then brought into contact with the PBS buffer solution, and the interface was probed through the backside of the prism. A 0.1 mg/ml protein solution in 1× PBS buffer and *p*H 7 was injected into the flow cell, replacing the clean buffer inside the cell.

### E. SFG spectra calculations

SFG spectra were calculated using the method described in Ref. 41. From the atom coordinates in the protein structure files from the Monte Carlo simulations, we determined the couplings between the amide groups: nearest neighbor couplings are calculated using *ab initio* methods that give the coupling as a function of the dihedral angle between the neighboring amide moieties; non-nearest neighbor couplings are calculated with a coulomb-like transition dipole coupling model. After diagonalizing the Hamiltonian, we calculated the IR and Raman modes of the protein from the eigenvalues and eigenvectors, and then take their outer product to calculate the vibrational SFG response. The background phase was kept at 3.5 and 1.5 rad for ssp (s-polarized SFG, s-polarized visible, and p-polarized IR) and ppp, respectively, which gave the best results, and thus these values were used for all spectra calculations.

### F. Materials

Recombinant protein G B1 was expressed in *Escherichia coli* and purified using IMAC and the SMT3/ULP1 protease to cleave the 10xHis tag, then verified for identity using intact mass spectrometry. Protein G B1 for the SFG experiments was expressed and purified as described elsewhere.^42^ IgG antibody and PBS solution (0.01 M phosphate, 0.138 M sodium chloride, and 0.0027 M potassium chloride, *p*H 7.4) was purchased from Sigma (Sigma-Aldrich, St. Louis, MO).

## III. RESULTS

### A. Testing the Monte Carlo algorithm using the LKα14 peptide

**Fig. 1.**
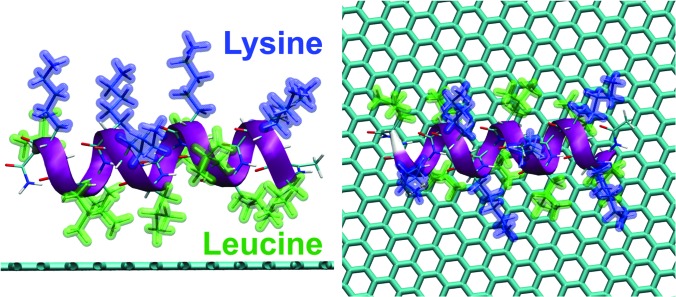
Two different views of the LKα14 peptide on a graphene surface.

The LKα14 peptide, which consists of only leucine and lysine residues (Fig. 1), was used as a benchmark because its orientation on hydrophobic surfaces has been determined experimentally in previous studies.^43–46^ When adsorbed onto either a charged or hydrophobic surface, this peptide assembles into an alpha-helical secondary structure where all the leucine residues lie on one side of the alpha-helix and all the lysine residues lie on the other side.^44^ Thus, the LKα14 peptide has a predictable conformation and orientation on hydrophobic surfaces, such as polystyrene and methyl-terminated self-assembled monolayers, and its orientation and structure has been extensively studied with a wide variety of experimental and simulation techniques, including solid state NMR, SFG, and MD.^40,44,45,47^ This makes LKα14 on graphene a good benchmark system for testing the Monte Carlo method for protein orientation studies.

**Fig. 2.**
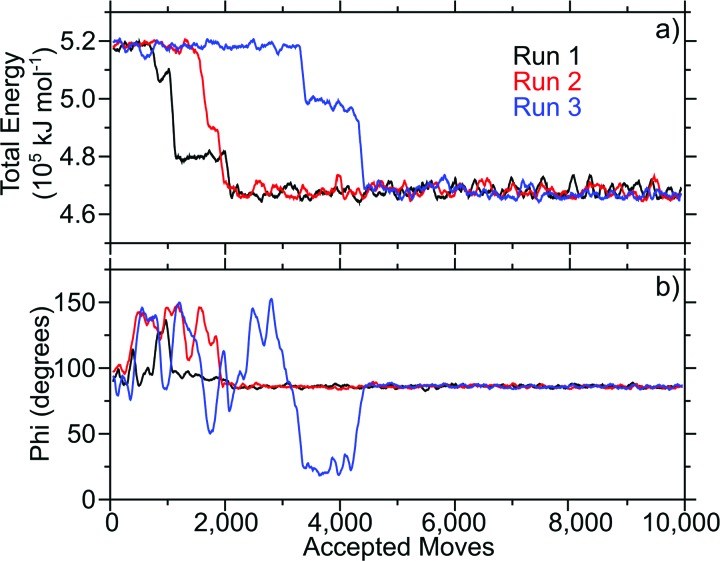
Total energy (a) and principal axis angle (b) of the LKα14 on graphene over the course of 10 000 accepted moves in three Monte Carlo simulations (shown in black, red, and blue). The simulations were started with the LKα14 peptide oriented such that the lysine residues were closest to the graphene surface. All plots show a 100-pt running average.

For this purpose, three simulations of 10 000 accepted moves each were started with the LKα14 peptide near the graphene surface. The LKα14 peptide was generated using vmd (Ref. 30) and each of the simulations started with the lysine amino acid residues closest to the surface. By the end of all three simulations, the LKα14 peptide flipped its orientation with the leucine amino acid residues closest to the surface. The total energy was monitored along the trajectories [Fig. 2(a)]. The time series of the total energy revealed that the three simulations converged to one minimum. Previous SFG studies suggested an average orientation angle between the peptide backbone and the surface normal to be ∼80°, or almost parallel to the surface.^48^ In its most frequently sampled orientation, the angle between the principal axis of the peptide and the surface normal vector for the MC simulations was ∼86° [Fig. 2(b)], consistent with the SFG results. The agreement between the Monte Carlo and SFG results suggest that the Monte Carlo algorithm developed in this study can correctly describe the orientation of LKα14 adsorbed onto graphene surfaces.

### B. Predicting the orientation of protein G B1 on a graphene surface

**Fig. 3.**
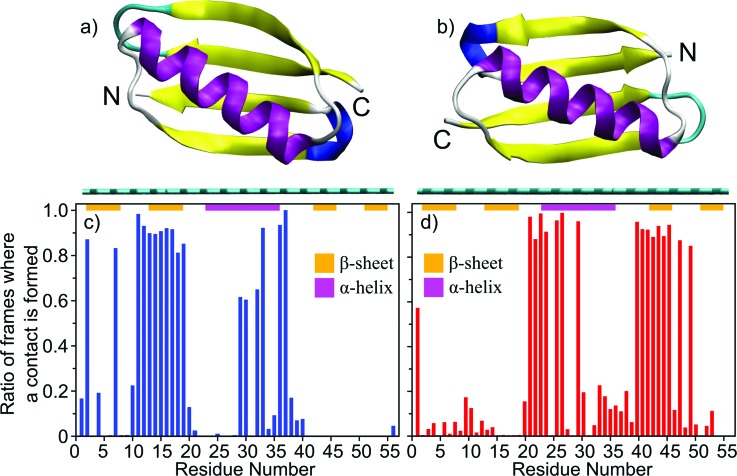
Predicted orientations of protein G B1 on a graphene surface through Monte Carlo simulations. The runs converged toward either the orientation represented in (a) or the orientation represented in (b). The graphene surface is colored in cyan and it is visible below the protein. The N- and C-termini of the protein are labeled. (c) and (d) Ratio of accepted Monte Carlo moves (referred to here as frames) where a particular residue is within 6 Å from the surface in the runs that converged either toward the orientation in (a) or in (b), respectively. The ratio is calculated with respect to the total number of sampled orientations where any atom of the protein is within 6 Å from the surface [prior to the calculation, the frames from all runs that converged toward either the orientation in (a) or in (b) were merged]. In the plots, the secondary structure regions are indicated with horizontal bars and colored in magenta for α-helices or orange for β-sheets, respectively.

The Monte Carlo algorithm was applied to a more complex, but widely studied, IgG antibody-binding domain of protein G, protein G B1. This 6 kDa binding domain consists of 56 residues and, when in solution, it adopts a secondary structure consisting of two β-sheets and one α-helix [Figs. 3(a) and 3(b)]. Like with the LKα14 peptide, Monte Carlo simulations were performed with protein G B1 on a graphene surface.

**Fig. 4.**
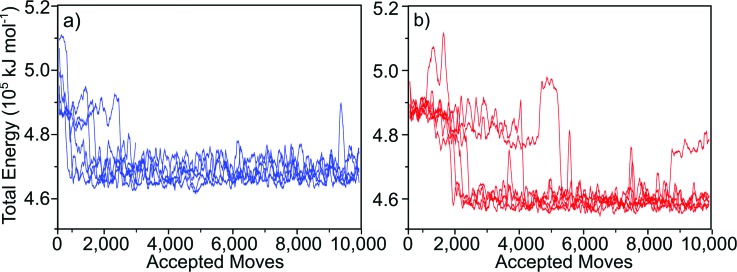
Total energy of the system over the course of 10 000 accepted moves. The two minimums were reached an equal number of times. (a) Orientation predicted in runs 1, 3, 5, 8, 9, and12. (b) Orientation predicted in runs 2, 4, 6, 7, 10, and 11. All plots show 100-pt running average.

In total, 13 MC simulations were run using the same starting conformation. In one of the 13 simulations, the protein moved away from the surface and did not return during the course of the simulation, so this run was discarded. In the remaining 12 simulations, the protein converged to a stable orientation. The total energy was monitored along the trajectories. The time series of the total energy (Fig. 4) revealed that each of the remaining 12 simulations converged to one of two energy minima, which corresponded to two distinct orientations of protein G B1 on the surface [Figs. 3(a) and 3(b)]. Runs 1, 3, 5, 8, 9, and 12 converged to one [Figs. 3(a) and 3(c)] while runs 2, 4, 6, 7, 10, and 11 converged to the other minimum [Figs. 3(b) and 3(d)].

The visual analysis of the PDB structures corresponding to the minima revealed that part of the α-helix and either one of the outermost β-strands contact the surface [Figs. 3(a) and 3(b)]. The predicted orientations differ from each other by a rotation of ∼180° around the second largest principal axis of the protein [Figs. 3(a) and 3(b)]. This can be quantitatively described by calculating the ratio of frames where a particular residue is in contact with the surface with respect to the total number of frames where the protein contacts the surface in simulations that converged toward either minimum [Figs. 3(c) and 3(d)]. Residues 13–19 in Fig. 3(c) and residues 42–46 in Fig. 3(d) correspond to the outermost β-strands contacting the graphene surface in the two predicted orientations, respectively [Figs. 3(a) and 3(b)]. Residues 23–36 correspond to the α-helix, part of which contacts the surface in both orientations. This can be seen from the large number of interactions in either the N- [Fig. 3(c)] or the C-terminal part [Fig. 3(d)] of the α-helix, respectively [Figs. 3(a) and 3(b)].

**Fig. 5.**
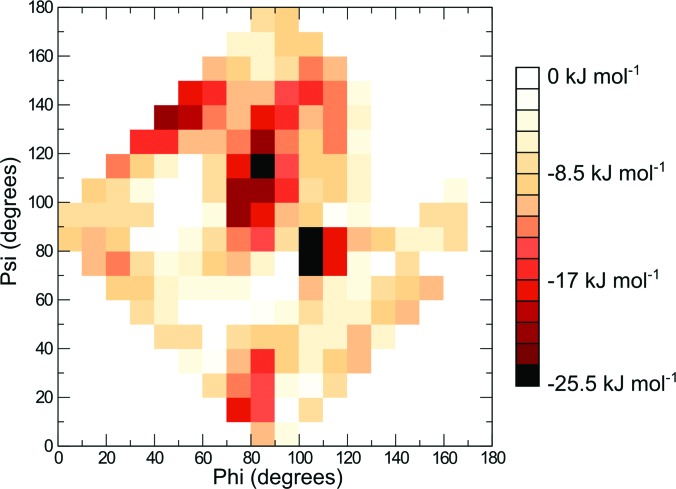
Free energy heatmap for all 12 simulated Monte Carlo runs of protein G B1 on graphene. Ranges of 10° are shown.

To further quantify the two predicted orientations of protein G B1 on graphene, a free energy heatmap was created using as collective variables the angles (phi and psi) between the two major principal axes and the surface normal vector (supplementary Fig. S1).^65^ The free energy was calculated by taking the negative natural logarithm of the number of times a particular orientation was sampled and multiplying it by the thermal fluctuations, G = −kT ln (N), where N is the count for each orientation (Fig. 5). Two free energy minima were identified: phi = 80–90 and psi = 110–120; phi = 100–110 and psi = 70–90 (Fig. 5). These minima corresponded to the two orientations toward which the simulations converged [Figs. 3(a) and 3(b), respectively].

### C. Verification of the assumption that protein G B1 can be treated as a rigid body

**Fig. 6.**
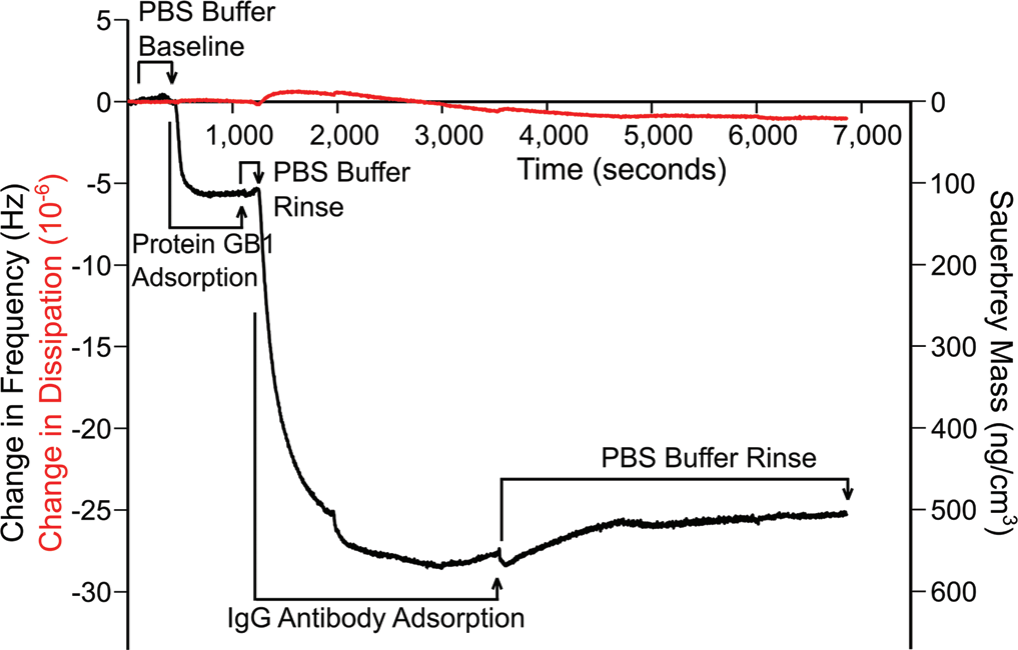
Changes in frequency and Sauerbrey mass (black) and dissipation (red) as a function of time during the adsorption of protein G B1 onto a polystyrene surface followed by binding of IgG antibody to the adsorbed protein G B1. After a buffer baseline was established, 5 *μ*g/ml protein G B1 in PBS was introduced for 13 min and followed by a buffer rinse. Then, 5 *μ*g/ml IgG antibody was introduced for 39 min and followed by a final buffer rinse.

One of the assumptions we have used in the development of the Monte Carlo simulations is that the protein is rigid and does not undergo significant conformational changes upon adsorption onto hydrophobic surfaces. To experimentally validate this assumption, we utilized quartz crystal microbalance with dissipation monitoring. This technique monitors the amount of protein immobilized onto a piezoelectric quartz crystal and how the viscoelasticity/rigidity of the proteins changes upon immobilization. In the QCM-D study, protein G B1 was adsorbed onto hydrophobic polystyrene-coated quartz crystals. The frequency (F) and dissipation (D) during protein G B1 adsorption was monitored, and no significant changes in dissipation were detected (Fig. 6), i.e., the ratio of ΔD/ΔF was <0.05 as protein G B1 was adsorbed. This suggests that no major conformational changes in protein G B1 occurred during the adsorption process.^49–51^ We also examined the binding of IgG antibody to the immobilized layer of protein G B1 to determine whether, when immobilized, protein G B1 was still capable of binding IgG. From the frequency shifts, it was determined using the Sauerbrey equation^52^ that 98 ng/cm^2^ of protein G B1 was adsorbed onto the polystyrene surface and 350 ng/cm^2^ of IgG bound to the protein G B1 covered surface. This confirms that protein G B1 adsorbed onto the hydrophobic polystyrene surface maintains IgG antibody-binding activity.

### D. Verification of predicted orientations through sum frequency generation experiments

SFG spectroscopy was used to experimentally test whether the MC simulations predicted realistic orientations of adsorbed protein G B1. For SFG vibrational spectroscopy, a visible laser beam and a tunable infrared beam are overlapped in time and space at the surface. The sum frequency signal, which is generated at the interface due to nonlinear optical frequency mixing, is enhanced and yields a peak in the spectrum, when interfacial species are in resonance with the infrared light.^53^ SFG has, over the past years, been developed into a reliable tool to determine protein folding and orientation on surfaces *in situ*.^48^ Owing to the nonlinear optical selection rules of SFG, only ordered protein layers at the interface are detected. Unbound and disordered proteins, even if close to the surface in solution, cannot not detected. The spectra therefore only represent the ordered proteins within the ensemble of proteins present at the surface. In analogy to Raman or infrared spectroscopy, the amide I modes can provide detailed information about the folding and structure of interfacial proteins.

**Fig. 7.**
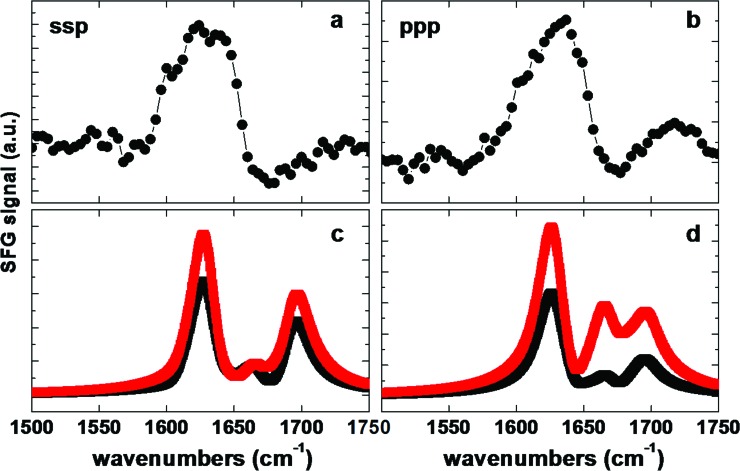
Experimental and calculated SFG spectra. [(a) and (b)] Experimental spectra for protein G B1 on polystyrene in ssp and ppp polarization combination. [(c) and (d)] Spectra calculated from the first predicted orientation shown in Fig. 3(a) (red) and from the second predicted orientation shown in Fig. 3(b) (black).

Amide I SFG spectra of protein G B1 adsorbed onto polystyrene surfaces are shown in Figs. 7(a) and 7(b). The spectra are related to two different polarization combinations: ssp (s-polarized SFG, s-polarized visible, and p-polarized IR) and ppp. The ssp spectra exhibit positive peaks near 1601, 1623, and 1639 cm^−1^ and a negative peak near 1675 cm^−1^. The ppp spectra contain positive features near 1609, 1634, and 1722 cm^−1^. The modes near 1630 and 1675 cm^−1^ are typically assigned to ordered β-sheet structures.^24,42,54^ The ppp feature near 1722 cm^−1^ is likely related to side chain modes.^54^ The low frequency modes below 1610 cm^−1^ are difficult to assign and could be related to side chain modes or caused by interferences of several delocalized backbone modes. The challenges involved with peak assignment in SFG spectra can be traced back to the extensive interference between modes within a complex protein. This is an important difference to linear vibrational spectroscopies, where different adjacent modes overlap but do not influence each other. In SFG, vibrational modes will interfere, which leads to complex spectral shapes.^55^ To extract structural information from such spectra, theoretical spectra can be calculated from protein structure files^56^ and combined with experimental SFG data.^41,57–59^

Theoretical SFG spectra were calculated using each of the two adsorbed orientations of protein G B1 determined by the MC simulations [Figs. 7(c) and 7(d)]. Clearly, the calculated spectra do not capture the spectral features of the experimental data well. In ssp, while the calculated spectra capture the main resonance near 1630 cm^−1^, they also produce an unexpected feature near 1700 cm^−1^ [Figs. 7(a) and 7(c)]. In ppp polarization, the calculations reproduce the main peak near 1630 cm^−1^ but miss any low frequency shoulders and produce two high-energy modes, which are not visible in the experimental data [Figs. 7(b) and 7(d)]. It should be noted that tests with several other orientations yielded no improvements in the agreement between theory and experiment.

**Fig. 8.**
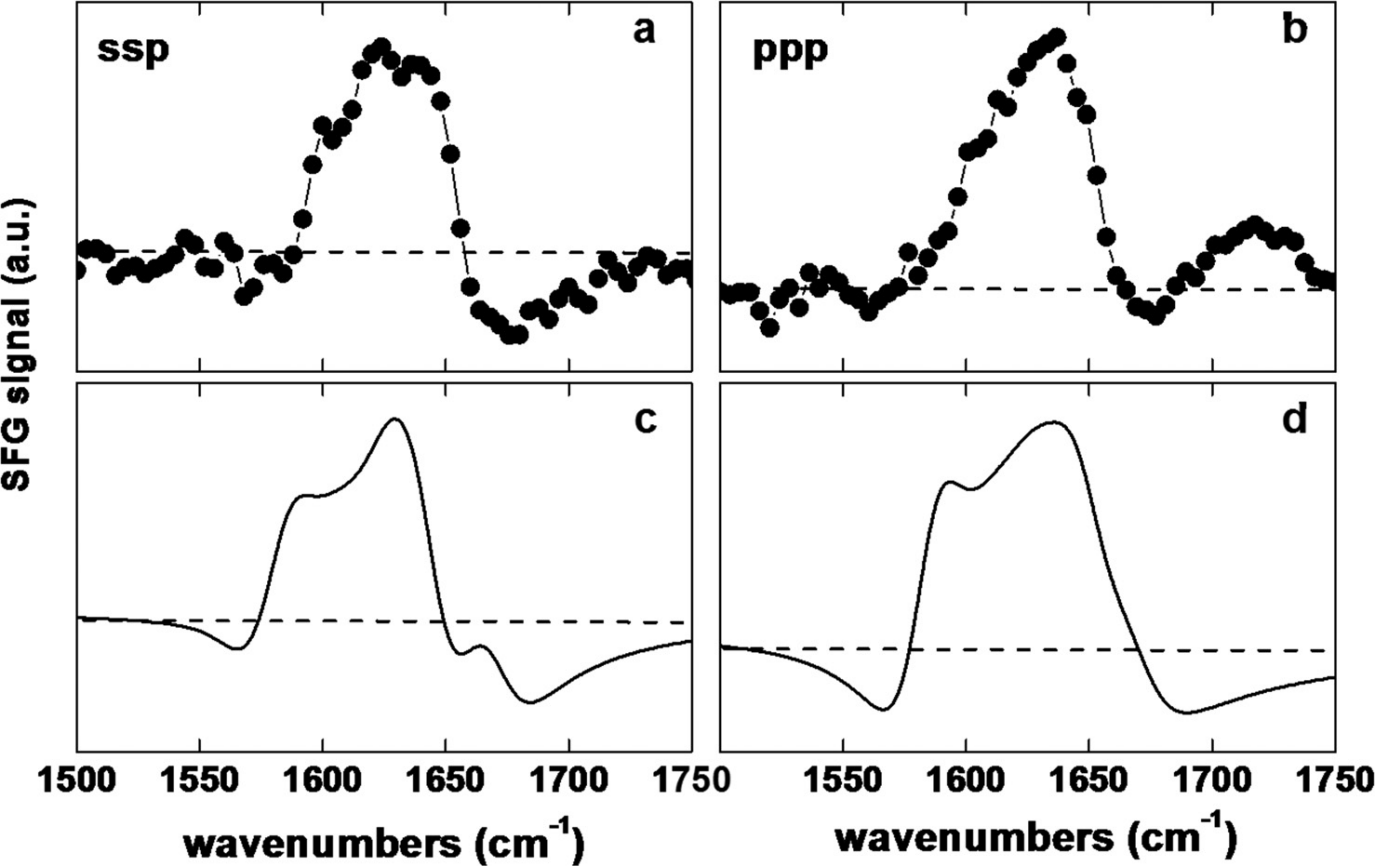
Experimental and calculated SFG spectra. [(a) and (b)] Experimental spectra for protein G B1 adsorbed onto polystyrene in the ssp and ppp polarization combinations. [(c) and (d)] Spectra calculated for a 50/50 mixture of the two predicted orientations.

Since the two orientations predicted by the MC simulations are close in energy it is likely that both orientations will be present on the surface. We therefore calculated the SFG response of a monolayer of protein G B1 with a 50/50 mixture of both orientations. When using a 50/50 mixture of both orientations for the spectra calculations, the peaks around the main feature near 1630 cm^−1^, including the low energy features, as well as the negative feature near 1675 cm^−1^, are now captured by the theoretical spectra (Fig. 8). Therefore, the results of the calculations match the experimental data better than either of the two orientations individually.

### E. Stability of the protein G B1 predicted orientations in MD simulations

**Fig. 9.**
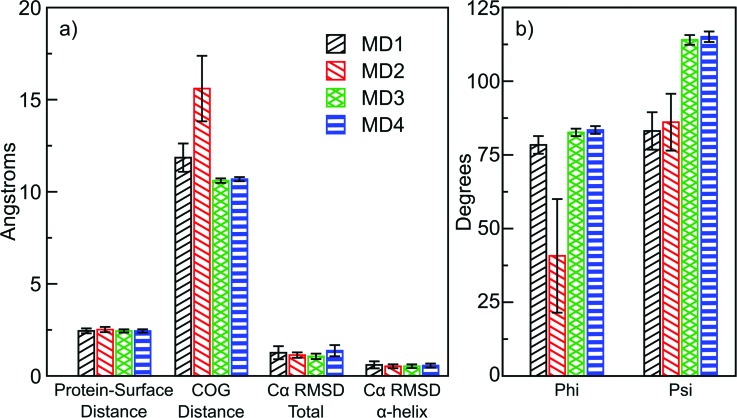
Averages of quantities measured during the molecular dynamics simulations started from the Monte Carlo predicted orientations of protein G B1 on the graphene surface. The bars show a time average along the simulations, while error bars represent the standard deviation.

To test the conformational stability of protein G B1 adsorbed onto a graphene surface, four 50-ns long MD simulations in explicit water were started from the predicted orientations of protein G B1. Two of the simulations (MD1 and MD2) were started from one of the predicted orientations [Fig. 3(b)] and two (MD3 and MD4) from the other predicted orientation [Fig. 3(a)]. In all four simulations, the protein remained within 2.5 Å from the surface [Fig. 9(a)]. However, in MD2, the protein tilted to an orientation with one of its smaller sides contacting the surface and the rest of it solvent exposed. This is indicated by its relatively larger average distance between the center of geometry (COG) and the surface [Fig. 9(a)] and the smaller angle Phi between its major axis and the normal to the surface [Fig. 9(b)]. In the other three runs, the protein maintained its initial orientation throughout the entire simulation length, as indicated by the similar distance between the COG and the surface [Fig. 9(a)] and the similar angles Phi and Psi between its two major axes and the normal to the surface [Fig. 9(b)]. Generally, the protein maintained its three-dimensional structure and the α-helix was conserved throughout the simulations as indicated by their backbone Cα atom root mean square deviations from the initial conformation [Fig. 9(a)].

Interestingly, a slight distortion was observed in MD1 where the α-helix briefly tilted outward away from the rest of the protein while generally maintaining its secondary structure but returned to its native position after ca. 12 ns (supplementary Fig. S2).^65^ It is plausible that protein G B1 might undergo slight adjustments of its three-dimensional structure upon adsorption to a hydrophobic surface. However, this was an isolated and short-lived event, and thus, no strong conclusions can be drawn. The fact that the protein tilted in one out of four runs could be an artifact of the force field used in the simulations and current force fields might need to be optimized for protein–surface simulations,^60,61^ or it could indicate that the protein probes alternative but less populated orientations upon adsorption. It would be interesting to investigate these events in longer time-scale MD simulations in a future study. It also needs to be noted that while the experiments were performed with polystyrene, the simulations were performed with graphene. This does not affect the Monte Carlo calculations since the protein is treated as a rigid body and both surface types are hydrophobic. However, one might want to consider using a polystyrene surface for long time-scale MD simulations.

## IV. DISCUSSION

Determining the orientation and structure of proteins adsorbed onto the surface of materials is challenging because although well-established surface analysis methods such as ToF-SIMS provide important details about adsorbed proteins, they do not provide structural information at the atomic level. It would be desirable to have methods that directly determine the composition of biofilms at atomic level of detail, as it is, for example, already the case for proteins in solution and crystals. Because of this technological gap, there is a need for computational tools that can complement and extend experimental surface analysis techniques by providing atomistic details about the protein adsorption process.^48^ Computational predictions can then be validated through experimental measurements, and the combination of computations and experiments can provide a detailed and realistic model about the structure of protein films.

Here, a Monte Carlo based algorithm was developed by combining existing molecular dynamics simulation and visualization software with a SASA based implicit solvation model to determine the orientation of protein G B1 adsorbed onto hydrophobic surfaces. The simulations converged toward two distinct orientations (Fig. 3) that corresponded to two minima with roughly equal free energy values (Fig. 5). Strikingly, SFG experiments confirmed that protein G B1 indeed has a roughly 50% probability of being found in either one of the two predicted orientations on a polystyrene surface (Fig. 8). This highlights the importance of complementing current surface analysis techniques with atomistic computational predictions, since, as shown in this case, it would have been hard to uniquely determine from SFG data alone the number of orientations that were present. Finally, MD simulations in explicit water illustrated how the MC-based method developed here can be used to efficiently generate a starting system for studying conformational fluctuations of adsorbed proteins.

The atomistic understanding of the protein adsorption process can provide insight how different surface materials can differentially influence the function of proteins. For example, recently it has been shown that the coagulation protein von Willebrand factor (VWF) is activated when adsorbed on a polystyrene surface but inactive on a glass surface.^62^ A combination of Monte Carlo and MD simulations as presented here could help discriminate whether this is due to different orientations or to conformational changes of VWF on a hydrophobic versus a hydrophilic surface. It needs to be noted that the method presented here does not take into account that protein molecules can also interact with each other besides with the surface and that this could influence their orientation or conformation. However, since the polypeptides used here are soluble, it is likely that during the adsorption process the protein–surface interaction will be more dominant than any interprotein interactions. Consistent with this, in a recently published simulation, the LK peptide was observed to assume a similar surface orientation whether it was isolated or whether another LK peptide was present.^63^ Nonetheless, more complex algorithms could be developed to take interprotein interactions into account for cases where these effects are suspected to play a major role in the system under study.

In the future, it will be necessary to also predict protein orientations on hydrophilic surfaces. Unlike in the case of nonpolar surfaces such as graphene, electrostatic interactions need to be accounted for when studying adsorption onto hydrophilic surfaces. Since the energy evaluation in the Monte Carlo algorithm is based on an implicit solvation model, screening effects of the water will also have to be considered, either by using a constant or a distance-dependent dielectric coefficient. This is challenging because distance-dependent dielectric constants are known to overestimate electrostatic interactions between charged side chains in protein folding studies with implicit solvent models.^29,64^ Another challenge is that more advanced implicit solvation models based on Generalized Born^31,32^ are not adequate to study the interaction between molecules and surfaces. Finally, in cases of surfaces that are known to order water, it might be necessary to include water molecules explicitly. Overall, the development of better implicit solvation models along with the implementation of Monte Carlo algorithms to predict the orientation of proteins on surfaces presents an interesting and exciting challenge.

## V. CONCLUSIONS

The following conclusions can be drawn from this work:

(1) The Monte Carlo simulations predicted two distinct orientations of protein G B1 on hydrophobic graphene surfaces.
(2) The QCM-D results indicated that upon adsorption onto polystyrene surfaces protein G B1 did not undergo major conformational changes and maintained its IgG antibody binding activity. This justifies the assumption that the protein can be treated as a rigid body in Monte Carlo simulations.
(3) Calculated SFG spectra based on a 50/50 mixture of the two Monte Carlo predicted orientations agreed with experimental data of protein G B1 adsorbed onto polystyrene confirming that an ensemble containing at least the two predicted orientations was present on the surface.
(4) In MD simulations started from the predicted Monte Carlo orientations, the protein was generally stable, remained in contact with the surface, and in three out of four runs it maintained the predicted orientation.

The Monte Carlo simulations developed here can provide atomic-level detail of protein–surface interactions that are not typically available from experimental surface analysis techniques. The predicted orientations can be used to complement results from surface analysis experiments and as starting structure for more accurate explicit solvent molecular dynamics simulations to study conformational changes of the protein at the protein–surface interface.
